# Supplementary material for: Profiles Combining Muscle Atrophy and Neutrophil-to-Lymphocyte Ratio Are Associated with Prognosis of Patients with Stage IV Gastric Cancer
Source: Nutrients. 2020 Jun 24;12(6):1884. doi: 10.3390/nu12061884 (PMC7353220; doi:10.3390/nu12061884)
Supplement: Supplementary file 1 [file nutrients-12-01884-s001.pdf]

**Supplementary Table S1.** Biochemical parameters or enrolled subjects.

| | Reference Value | Median (IQR) | Range (min–max) |
|---|---|---|---|
| Red blood cell count (×10⁴/μL) | 435–555 | 389 (366–439) | 253–552 |
| Hemoglobin (g/dL) | 13.7–16.8 | 11.2 (9.8–12.7) | 6.9–16.5 |
| White blood cell count (/μL) | 3300–8600 | 7000 (3400–8600) | 3200–18,000 |
| Platelet count (x10³/mm³) | 15.8–34.8 | 274 (223–375) | 127–622 |
| AST (IU/L) | 13–30 | 25 (18.5–38) | 8–315 |
| ALT (IU/L) | 10–30 | 18 (12–28) | 4–277 |
| LDH(IU/L) | 124－222 | 212 (168.5–343.5) | 106-2,323 |
| ALP (IU/L) | 115–359 | 284 (223.5–479.5) | 78–3,911 |
| GGT (IU/L) | 13–64 | 33 (17–108) | 9–1,071 |
| Total protein (g/dL) | 6.6–8.1 | 6.78 (6.4–7.21) | 4.95–8.1 |
| Albumin (g/dL) | 4.1–5.1 | 3.5 (3.12–3.86) | 1.8–4.85 |
| Prothrombin activity (%) | 80–120 | 98.5 (88.7–107) | 21.7–130 |
| Total bilirubin (mg/dL) | 0.40–1.20 | 0.51 (0.41–0.7) | 0.25–4.64 |
| Total cholesterol (mg/dL) | 142–219 | 169 (142.8–199) | 94–291 |
| Triglyceride (mg/dL) | 40–149 | 96 (75–121) | 41–314 |
| BUN (mg/dL) | 8.0–20.0 | 13.4 (11.6–18.0) | 7–164 |
| Creatinine (mg/dL) | 0.65–1.07 | 0.77 (0.64–0.94) | 0.32–6.15 |
| eGFR (mL/min/1.73 $m^2$) | >90.0 | 72.4 (60.7–85.2) | 6.1–141.3 |
| Blood glucose (mg/dL) | 80–109 | 105.5 (97–125) | 72–288 |
| HbA1c (%) | 4.3–5.8 | 5.9 (5.5–6.2) | 3.9–10.9 |
| CEA | ≤ 5.0 | 8.5 (3.5–32.6) | 0.3–2,868 |
| CA19-9 | ≤ 37.0 | 24.4 (7.05–95.9) | 1–109,278 |

Note: Data are expressed as median (interquartile range [IQR]), range, or frequency. Abbreviations: IQR, interquartile range; AST, aspartate aminotransferase; ALT, alanine aminotransferase; LDH, lactate dehydrogenase; ALP, alkaline phosphatase; GGT, gamma-glutamyl transpeptidase; BUN, blood urea nitrogen; eGFR, estimated glomerular filtration rate; HbA1c, hemoglobin A1c; CEA, carcinoembryonic antigen.

**Supplementary Table S2.** Comparison of biochemical parameters between the Alive and Deceased groups.

| | Alive | | Deceased | | |
|---|---|---|---|---|---|
| | **Median (IQR)** | **Range (Min–Max)** | **Median (IQR)** | **Range (Min–Max)** | ***p*** |
| Red blood cell count (×10⁴/μL) | 389 (354–430) | 270–552 | 389 (367–443) | 253–528 | 0.6102 |
| Hemoglobin (g/dL) | 11.2 (9.8–12.3) | 7.0–15.3 | 11.4 (9.8–12.7) | 6.9–16.5 | 0.9089 |
| White blood cell count (/μL) | 6,700 (5,600–8,100) | 4,000–13,600 | 7,100 (6,200–8,700) | 3,200–18,000 | 0.2195 |
| Platelet count (x10³/mm³) | 261 (223–372) | 154–462 | 280 (221–375) | 127–622 | 0.7366 |
| AST (IU/L) | 22 (18.3–34.5) | 12–56 | 26 (18.5–38.5) | 8–315 | 0.6271 |
| ALT (IU/L) | 20 (13.3–25.8) | 5–48 | 17 (11–32) | 4–277 | 0.8759 |
| ALP (IU/L) | 269 (212–374) | 148–1,262 | 293 (224–541) | 78–3,911 | 0.3617 |
| GGT (IU/L) | 29 (18–66) | 10–365 | 37 (16–119) | 9–1071 | 0.6396 |
| Total protein (g/dL) | 6.99 (6.43–7.49) | 5.10–8.10 | 6.75 (6.39–7.70) | 4.95–8.00 | 0.1561 |
| Prothrombin activity (%) | 95 (86–103) | 22–127 | 99 (92–108) | 54–130 | 0.1203 |
| Total bilirubin (mg/dL) | 0.50 (0.40–0.70) | 0.25–3.40 | 0.55 (0.42–0.71) | 0.25–4.64 | 0.6037 |
| Total cholesterol (mg/dL) | 166 (136–187) | 111–235 | 169 (147–206) | 94–291 | 0.1747 |
| Triglyceride (mg/dL) | 93 (70–122) | 54–149 | 99 (75–121) | 41–314 | 0.6798 |
| BUN (mg/dL) | 13.7 (11.5–20.8) | 7–35.1 | 13.4 (11.6–17.8) | 8–164 | 0.7734 |
| Creatinine (mg/dL) | 0.79 (0.68–0.94) | 0.49–5.96 | 0.75 (0.63–0.94) | 0.32–6.15 | 0.4051 |
| eGFR (mL/min/1.73 m²) | 71.3 (62.9–83.5) | 6.1–103.6 | 72.4 (60.5–89.6) | 7.7–141.3 | 0.5485 |
| Blood glucose (mg/dL) | 104 (95–111) | 72–158 | 108 (100–132) | 79–288 | 0.1219 |
| CEA | 8.0 (3.2–19.3) | 0.7–345 | 8.5 (3.7–55.2) | 0.3–2,868 | 0.4580 |
| CA19-9 | 17.1 (5.4–68.8) | 2–109,278 | 27.0 (7.6–111.5) | 1–88,806 | 0.3709 |

Note: Data are expressed as median (interquartile range [IQR]), range, or frequency. Abbreviations: IQR, interquartile range; SOX, S-1 with oxaliplatin; SP, S-1 with cisplatin; FOLFOX, 5-fluorouracil with oxaliplatin; Cape, capecitabine; 5-FU, 5-fluorouracil; SOXT, S-1 with oxaliplatin and trastuzumab; SPT, S-1 with cisplatin and trastuzumab; CAPOXT, capecitabine with oxaliplatin and trastuzumab; XPT, capecitabine with cisplatin and trastuzumab; tub, tubular adenocarcinoma; por, poorly differentiated adenocarcinoma; sig, signet-ring cell carcinoma; muc, mucinous adenocarcinoma; AST, aspartate aminotransferase; ALT, alanine aminotransferase; LDH, lactate dehydrogenase; ALP, alkaline phosphatase; GGT, gamma-glutamyl transpeptidase; BUN, blood urea nitrogen; eGFR, estimated glomerular filtration rate; HbA1c, hemoglobin A1c; CEA, carcinoembryonic antigen; NLR, neutrophil-to-lymphocyte ratio.
